# Systemic antisense therapeutics inhibiting DUX4 expression ameliorates FSHD-like pathology in an FSHD mouse model

**DOI:** 10.1093/hmg/ddab136

**Published:** 2021-05-13

**Authors:** Ngoc Lu-Nguyen, Alberto Malerba, Shan Herath, George Dickson, Linda Popplewell

**Affiliations:** Department of Biological Sciences, School of Life Sciences and the Environment, Royal Holloway University of London, Egham, Surrey TW20 0EX, UK; Department of Biological Sciences, School of Life Sciences and the Environment, Royal Holloway University of London, Egham, Surrey TW20 0EX, UK; Department of Biological Sciences, School of Life Sciences and the Environment, Royal Holloway University of London, Egham, Surrey TW20 0EX, UK; Department of Biological Sciences, School of Life Sciences and the Environment, Royal Holloway University of London, Egham, Surrey TW20 0EX, UK; Department of Biological Sciences, School of Life Sciences and the Environment, Royal Holloway University of London, Egham, Surrey TW20 0EX, UK

## Abstract

Aberrant expression of the double homeobox 4 (*DUX4*) gene in skeletal muscle causes muscle deterioration and weakness in Facioscapulohumeral muscular dystrophy (FSHD). Since the presence of a permissive pLAM1 polyadenylation signal is essential for stabilization of *DUX4* mRNA and translation of DUX4 protein, disrupting the function of this structure can prevent expression of DUX4. We and others have shown promising results using antisense approaches to reduce *DUX4* expression *in vitro* and *in vivo* following local intramuscular administration. Here we demonstrate that further development of the antisense chemistries enhances *in vitro* antisense efficacy. The optimal chemistry was conjugated to a cell-penetrating moiety and was systemically administered into the tamoxifen-inducible Cre-driver FLExDUX4 double-transgenic mouse model of FSHD. After four weekly treatments, mRNA quantities of *DUX4* and target genes were reduced by 50% that led to 12% amelioration in muscle atrophy, 52% improvement in *in situ* muscle strength, 17% reduction in muscle fibrosis and prevention of shift in the myofiber type profile. Systemic *DUX4* inhibition also significantly improved the locomotor activity and reduced the fatigue level by 22%. Our data demonstrate that the optimized antisense approach has potential of being further developed as a therapeutic strategy for FSHD.

## Introduction

Facioscapulohumeral muscular dystrophy (FSHD) is a rare autosomal dominant genetic disorder with an estimated prevalence of 1:20 000 (1). The disease is characterized by asymmetric atrophy and weakness of the muscles of the face, shoulders and upper arms, which is extended to the trunk and lower limbs (2). Despite being the third most common muscular dystrophy, there is no existing disease-modifying treatment available for FSHD. This is partly because the complex mechanism underlying the disease has not been fully elucidated, although many candidate genes for FSHD have been identified (3–8). Of these, aberrant expression of the double homeobox 4 (*DUX4*) retrogene in skeletal muscle has been suggested in numerous studies to be predominantly involved in the pathogenesis of FSHD (9–14).

In healthy individuals, the subtelomeric region of chromosome 4q35 has 11–100 D4Z4 macrosatellite repeats, with each D4Z4 unit containing an incomplete copy of *DUX4*; this encodes for the full open-reading frame of a transcription factor that is expressed exclusively in germline and early embryos but is silenced in adult somatic tissues, including muscle (15,16). In most FSHD patients, contraction of the D4Z4 array to 1–10 repeats, associated with hypomethylation of the region, allows transcription of *DUX4* from the terminal D4Z4 repeat (17). However, for the polyadenylation and stabilization of the *DUX4* transcript, and ultimately expression of the normally repressed DUX4 protein, the presence of a specific disease-permissive pLAM1 polyadenylation signal distal to the last D4Z4 unit is required (18). Inhibiting the activity of this structure leads to *DUX4* mRNA degradation through non–sense-mediated decay (19) and subsequently reduces protein synthesis. This has been achieved through the use of small interfering RNA (20), small hairpin RNA (21), microRNA (22), recombinant U7-small nuclear RNA (23) or antisense oligonucleotides (AONs) (20,24–26). Among these, AONs have several outstanding advantages. They can be administered without requirement of a viral vector, thereby avoiding triggering of an immune response (27). Their administration allows flexibility in dosage and frequency of administration to achieve the highest therapeutic efficacy while minimizing possible side effects. In addition, AONs can be conjugated to a cell-penetrating moiety to enhance therapeutic efficiencies (28). Importantly, antisense therapy has emerged as a viable clinical therapy since four AONs received conditional FDA approval for use in subsets of patients with Duchenne muscular dystrophy (DMD) (EXONDYS 51^®^, VYONDYS 53^®^ and VILTEPSO^®^) or spinal muscular atrophy (SPINRAZA^®^).

We and others have reported that AON strategies were effective in down-regulating *DUX4* expression in FSHD-derived myoblast cultures (20,24), in a xenograft mouse model carrying muscle biopsies from FSHD patients (29) and as a local intramuscular treatment in a recently developed FLExDUX4 mouse model of FSHD (26,30). Although these findings are promising, there remains lack of evidence of a systemic antisense effect; this is clinically important since a treatment for FSHD will need to suppress *DUX4* expression in a large number of skeletal muscles. Furthermore, expression of *DUX4* locally in the xenografted mice or at very low levels as seen in the FLExDUX4 model is not sufficient enough to recapitulate the phenotypes observed in FSHD patients (29,31).

Following promising *in vitro* work from ourselves and others (20,24,29), we have designed several AONs targeting both key elements in the 3′UTR of *DUX4* mRNA, the polyadenylation signal and the cleavage site. We initially investigated the antisense effect in FSHD-derived myoblasts and present here the enhanced efficacy of new sequences in down-regulating expression of *DUX4* and its downstream targets to levels detectable in healthy isogenic myoblasts. We further studied the systemic therapeutic benefit of the best performing AON in the tamoxifen-inducible Cre-driver FLExDUX4 double-transgenic mouse model, where *DUX4* is inducibly expressed to a pathogenic level (32). We observed over 50% reduction in mRNA quantities of *DUX4* and downstream genes with treatment that led to significant amelioration in the muscle atrophy, muscle strength, muscle histology and bodywide activity of treated mice. Our data suggest that this antisense strategy can be potentially developed as a therapeutic approach for FSHD.

## Results

### Newly designed antisense oligonucleotides significantly knockdown *DUX4* expression and ameliorate DUX4 pathology *in vitro*

We have previously shown that an antisense oligonucleotide (AON) with a phosphorodiamidate morpholino oligomer (PMO) backbone that targets the cleavage site (CS) of *DUX4* mRNA, PMO CS3, inhibited *DUX4* expression in FSHD myoblast cell cultures by over 50% (24). Another AON targeting the polyadenylation signal (PAS), located 20–30 nucleotides upstream of the *DUX4* CS, was also effective in down-regulating *DUX4* mRNA level by ~40% (24,29). Aiming to enhance the antisense efficacy against *DUX4*, we have designed four AONs that target both the PAS and CS, named PACS 1–4. Here, we assessed *DUX4* inhibitory effect of newly designed AONs relative to PMO CS3, considered as the positive PMO control. A PMO targeting the *HBB* mutation that causes β thalassemia was used as a negative PMO control (PMO SCR). Details of PMOs used are shown in Supplementary Material, Table S1.

**Figure 1:**
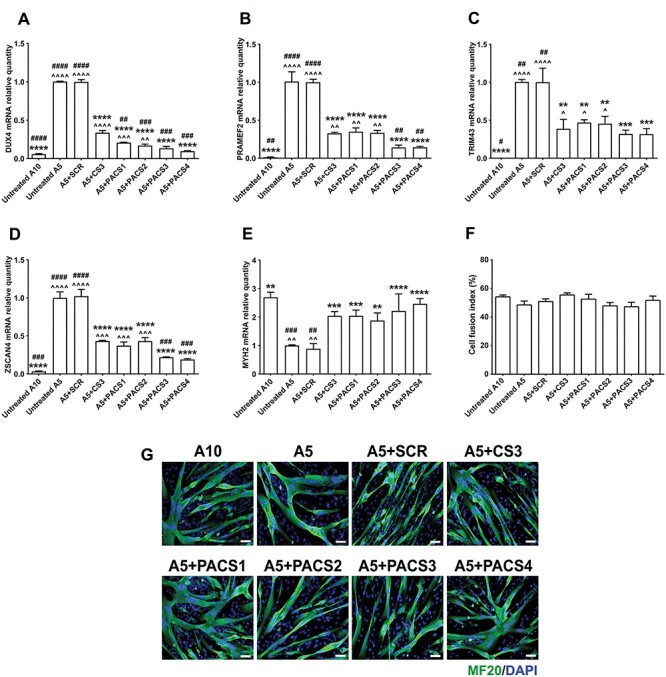
PMOs efficiently inhibit expression of *DUX4* and downstream targets in immortalized FSHD myoblast cell cultures. Immortalized A5 myoblasts were differentiated for 2 days before the cells were treated with 10 μm PMOs through Endo-Porter–mediated transfection. Immortalized A10 or A5 cells receiving only Endo-Porter reagent were considered as untreated positive or negative control, respectively. Total RNA was extracted two days after PMO treatment. RT-qPCR quantification for *DUX4* (**A**) and its targets: *PRAMEF2* (**B**), *TRIM43* (**C**), *ZSCAN4* (**D**) and a marker of cell differentiation, *MYH2* (**E**), are shown, relative to corresponding *B2M* expression. Cells in a parallel study were immunostained for all myosin isoforms using MF20 antibody. Cell fusion indexes were evaluated as the number of nuclei in MF20-positive myotubes containing ≥3 nuclei and expressed as the percentage of the total nuclei number in the image field (**F**). Representative cell images are displayed at magnification of ×100, scale bar = 100 μm, MF20 (green), DAPI (blue) (**G**). Statistical comparison (A–F) was by one-way ANOVA, followed by Tukey’s multiple comparisons test. Carets, asterisks or hashes indicate significances compared with untreated A10, untreated A5 or A5 treated with PMO CS3 (considered as positive PMO control), respectively. Data are shown as mean ± SEM, *n* = 3, *P* < 0.05 (^*^, ^, #), *P* < 0.01 (^*^^*^, ^^, ##), *P* < 0.001 (^*^^*^^*^, ^^^, ###), *P* < 0.0001 (^*^^*^^*^^*^, ^^^^, ####).

*In vitro* PMO screening was performed in FSHD-derived immortalized myoblast cells that have been characterized previously (33). The A5 clone containing three D4Z4 units represents patient cells, while the A10 clone containing 13 D4Z4 units represents healthy control. We induced differentiation to immortalized FSHD myoblasts for 2 days and then treated A5 cells with 10 μm of each PMO for 2 additional days. A5 and A10 cells receiving only the transfection reagent, Endo-Porter, were considered as untreated negative and positive controls, respectively (*n* = 3 per cell group). As shown in Figure 1, PMO SCR did not provide any inhibitory effect on the expression of any examined genes. Instead, all PACS PMOs were effective in down-regulating mRNA levels of *DUX4* and three examined genes, which have been identified to be predominantly up-regulated in FSHD cells and muscle biopsies (34,35), by 80–90% for *DUX4*, 65–85% for *PRAMEF2*, 53–68% for *TRIM43* and 57–81% for *ZSCAN4* (Fig. 1A–D). In comparison with PMO CS3, PMOs PACS1 and PACS2 further reduced *DUX4* expression by almost half of the level seen with CS3 treatment, although the inhibitory effect on expression of DUX4 target genes remained comparable to CS3. PMOs PACS3 and PACS4 were significantly and greatly more effective than CS3, completely down-regulating expression of all examined genes to the level of A10 positive control. The levels of expression displayed following PMO CS3 treatment remained significantly higher than the healthy values, specifically 6-fold for *DUX4* (*P <* 0.0001), 27-fold for *PRAMEF2* (*P* = 0.0040), 65-fold for *TRIM43* (*P* = 0.0479) and 13-fold for *ZSCAN4* (*P* = 0.0002).

Since inappropriate expression of *DUX4* and its downstream genes has been suggested to impair myotube formation (36), we further assessed the mRNA level of *MYH2* that is expressed by differentiated myotubes. All PMOs, except for PMO SCR, significantly improved the mRNA level of *MYH2* by 2–3-fold of the value in untreated A5, which was normalized to the healthy level of A10 control (Fig. 1E). However, we did not detect significant changes in the cell fusion index among all cell groups (*P* = 0.2408) (Fig. 1F); representative images of cells immunostained for a myosin marker are shown (Fig. 1G). This is consistent with previous findings (24,33) that report that contracted A5 cells, despite being isogenic, had higher fusion rate than the non-contracted A10 cells. Therefore, despite having significantly lower *MYH2* expression (*P* = 0.0025), the fusion index in untreated A5 cells was comparable with the level seen in A10 controls or in A5 cells receiving DUX4-targeting PMOs.

We also treated FSHD myoblasts with all PMOs at 1 μm. At this lower dose, newly designed PMOs remained effective in inhibiting expression of *DUX4* by 27–44%, *PRAMEF2* by 47–65%, *TRIM43* by 26–51% and *ZSCAN4* by 27–38%, although they were not significantly more efficient than PMO CS3 (Supplementary Material, Fig. S1a–d). PMO PACS4 continued to be the best performing candidate and was the only PMO that increased significantly the *MYH2* mRNA level relative to CS3-treated cells by 1.5-fold (*P* = 0.0167) (Supplementary Material, Fig. S1e). Based on these *in vitro* data, PMO PACS4 was significantly more effective than PMO CS3, the best candidate identified previously, in inhibiting expression of *DUX4-* and FSHD-related genes and therefore was selected for the *in vivo* study.

### Systemic antisense treatment efficiently improves the mass of several skeletal muscles in a tamoxifen-induced FSHD mouse model

To study the *in vivo* antisense effect, we used tamoxifen (TMX)-inducible Cre-driver FLExDUX4 double transgenic mice, named MCM-D4. Extensive characterization by the Jones Lab indicated that TMX-mediated induction can be either very mild or too severe depending on the dose regimen (31,32). Hence, to tailor the model for testing *in vivo* antisense treatment, we initially optimized the dose regimen of TMX for inducing *DUX4* expression. Sixteen-week-old male MCM-D4 mice were injected with either a single dose of 5 mg/kg TMX (*n* = 5) or 2.5 mg/kg/biweekly TMX (*n* = 5), dissolved in corn oil, via intraperitoneal (IP) administration. Age-matched HSA-MCM mice receiving volume-matched corn oil were considered as a positive control, CTRL (*n* = 5). The body weight recorded weekly (Supplementary Material, Fig. S2a) indicated a complete recovery of 100.9% start weight at week 4 following 5 mg/kg TMX induction, similar to 104.5% in CTRL mice (*P* = 0.0668), and consistent with the characterization by the Jones Lab (31,32). Although the same overall TMX dose was used, biweekly administration of 2.5 mg/kg TMX led to chronic weight loss by 12.5% (*P* < 0.0001) of the level seen in mice receiving a single dose of 5 mg/kg (Supplementary Material, Fig. S2a). Moreover, the mass of tibialis anterior (TA) correlated with the effect of TMX dosage with 26% (*P* < 0.0001) more muscle wasting observed following biweekly TMX injection than the single administration (Supplementary Material,Fig. S2b). *In situ* TA force measurement additionally demonstrated 30% (1015 ± 15.17 mN, *P* < 0.0001) and 65% (509 ± 69.21 mN, *P* < 0.0001) drop of CTRL force (1438 ± 40.45 mN) in mice injected with 5 mg/kg and 2.5 mg/kg/biweekly TMX, respectively (Supplementary Material, Fig. S2c). These data together suggested that the biweekly administration of 2.5 mg/kg TMX successfully generated a model with progressive DUX4-mediated muscle atrophy; therefore, this dose regimen was used to investigate our antisense approach *in vivo*.

**Figure 2:**
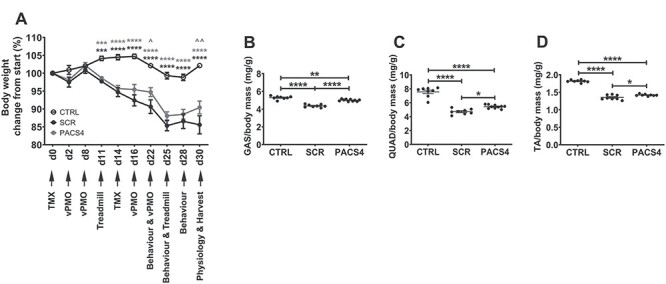
*In vivo* experimental plan and effect of Vivo-PMO PACS4 treatment on body and muscle mass. Details of the experiment, including timepoints for functional tests, and changes in body weight of mice are presented (**A**). All mice received 2.5 mg/kg/biweekly tamoxifen (TMX) by intraperitoneal (IP) injection. MCM-D4 mice were further IP injected with 10 mg/kg/week of Vivo-PMO PACS4 (PACS4, *n* = 5), or 10 mg/kg/week of Vivo-PMO SCR (SCR, *n* = 4), considered as a negative control. HSA-MCM mice receiving volume-matched saline were considered as positive control (CTRL, *n* = 4). Animals were sacrificed 1 week after the last Vivo-PMO injection. Muscle mass of the gastrocnemius—GAS (**B**), quadriceps—QUAD (**C**) and tibialis anterior—TA (**D**) normalized to the corresponding body weight is displayed; muscles on both sides of the body were used. Data are shown as mean ± SEM. Statistical analysis was by two-way (A) or one-way ANOVA (B–D) followed by Tukey’s multiple comparisons test, *P* < 0.05 (^*^, ^), *P* < 0.01 (^*^^*^, ^^), *P* < 0.001 (^*^^*^^*^), *P* < 0.0001 (^*^^*^^*^^*^). In (A), black asterisks indicate significance between SCR and CTRL groups; gray asterisks or carets indicate significance between PACS4 and CTRL or PACS4 and SCR groups, respectively.

PMO PACS4 was conjugated with an octaguanidine dendrimer chemistry (28), octaguanidine dendrimer-conjugated PMO (Vivo-PMO) PACS4, to enhance cell penetration. Sixteen-week-old male MCM-D4 mice were injected with 2.5 mg/kg TMX on days 0 and 14. Age-matched HSA-MCM mice receiving the same TMX dosage were considered as positive controls, CTRL (*n* = 4). MCM-D4 mice were further IP injected with 10 mg/kg of Vivo-PMO PACS4 (*n* = 5), or 10 mg/kg of Vivo-PMO SCR (*n* = 4), considered as negative control, on days 2, 8, 16 and 22, while HSA-MCM mice received volume-matched saline. Body weight recorded during the study, just prior to injections or functional tests, and normalized to the initial weight (Fig. 2A), displayed significant weight loss by ~6% in MCM-D4 versus CTRL mice on day 11 (*P* = 0.0023) that continued to drop by 12% (in PACS4, *P* < 0.0001) or 16% (in SCR, *P* < 0.0001) at the end of experiment. In comparison to SCR, treatment with PACS4 improved the body weight on day 22 by 4% (*P* = 0.0109) and remained effective at the end of the treatment (*P* = 0.0027). Accordingly with the observed body weight improvement, PACS4 administration significantly improved the mass of three examined muscles, including the gastrocnemius (GAS) from 4.37 ± 0.06 to 5.03 ± 0.04 mg/g (*P* < 0.0001), quadriceps (QUAD) from 4.72 ± 0.14 to 5.44 ± 0.08 mg/g (*P* = 0.0109) and tibialis anterior (TA) from 1.35 ± 0.02 to 1.42 ± 0.01 mg/g (*P* = 0.0189) (Fig. 2B–D). Thereby, PACS4 treatment significantly increased the mass of these muscles by up to 15% of the values of SCR-treated tissue.

### Systemic administration of Vivo-PMO PACS4 improves locomotor behavior, whole body function and TA muscle strength

**Figure 3:**
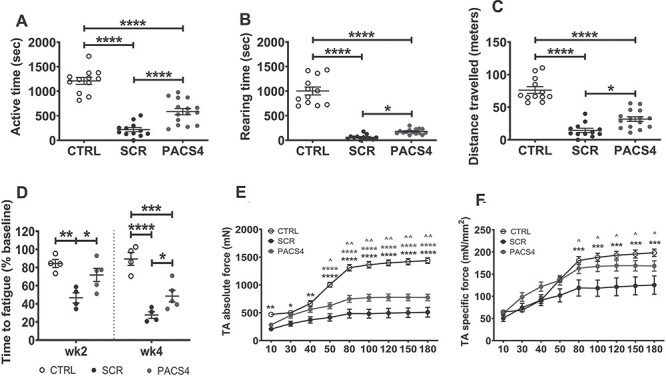
Systemic antisense administration improves animal behavior and muscle function. Mouse open-field behavior was assessed every 3 days for three times using locomotor activity monitors. Data obtained from each mouse/time were averaged. Representative parameters of the animal behavior are shown in (**A**–**C**), while details of all parameters are presented in Supplementary Material, Table S2. Effects of antisense therapy were further evaluated via treadmill exhaustion tests 1 week prior to the first TMX injection (baseline) and then at weeks 2 and 4 post-injection. Total running time on treadmill was recorded and expressed as the time to fatigue as the percentage of baseline time (**D**). Following four weekly Vivo-PMO administration, mice were put under terminal anesthesia and *in situ* absolute force of both TAs/mouse was measured (**E**). Specific muscle force is displayed as a ratio of the absolute force and the TA cross-sectional area (**F**). Data are shown as mean ± S.E.M, *n* = 4–5 mice. Statistical analysis was by one-way ANOVA (A–D) or two-way ANOVA (E, F) followed by Tukey’s multiple comparisons test, *P* < 0.05 (^*^, ^), *P* < 0.01 (^*^^*^, ^^), *P* < 0.001 (^*^^*^^*^), *P* < 0.0001 (^*^^*^^*^^*^).

After 4 weekly Vivo-PMO injections, we assessed the locomotor behavior of the animals using open-field activity cage monitors. MCM-D4 mice appeared less active than CTRL mice in 16 of 22 parameters examined (Supplementary Material, Table S2). However, following PACS4 treatment, MCM-D4 mice displayed a significant improvement in 11 parameters relative to mice receiving Vivo-PMO SCR. Notably, the total active time, total rearing time and travelled distance were all increased, from 215.4 ± 41.3 to 584.5 ± 64.0 s (*P* < 0.0001), 59.9 ± 14.3 to 182.6 ± 18.1 s (*P* = 0.0238), 14.6 ± 3.1 to 31.8 ± 3.6 m (*P* = 0.0116), respectively (Fig. 3A–C). We also evaluated the effect of antisense treatment on whole body function using treadmill exhaustion test. Mice were allowed to run on a treadmill until they were unable to move from the stopper for 10 s. The total running times were then calculated as the percentage of the baseline time recorded prior to the beginning of the experiment and are presented in Figure 3D, indicating that the time reaching exhaustion was significantly reduced in SCR-injected mice by 37% at week 2 (*P* = 0.0013) and 62% at week 4 (*P* < 0.0001), relative to CTRL group. However, mice receiving PACS4 displayed 27% (*P* = 0.0118) and 22% (*P* = 0.0249) less fatigue than SCR mice, respectively, and exhibited significant fatigue relative to CTRL group by 40% (*P* = 0.0007) only at week 4. Additional *in situ* TA force measurement demonstrated obvious muscle weakness in MCM-D4 mice receiving Vivo-PMO SCR, compared to CTRL group (Fig. 3E and F). Treatment with PACS4 significantly improved the maximal tetanic muscle force from 509.4 ± 87.2 to 773.8 ± 59.4 mN (*P* = 0.0049 at 180 Hz), relative to the SCR. Importantly, mice receiving PACS4 exhibited specific maximal muscle force comparable to CTRL mice, and that was significantly stronger than SCR-treated mice (125.4 ± 20.7 mN/mm^2^ versus 168.4 ± 11.6 mN/mm^2^, *P* = 0.0351 at 180 Hz). The data together suggest that Vivo-PMO PACS4 administration slowed down the disease progression in the treated mice.

### Vivo-PMO PACS4 robustly inhibits mRNA expression of *DUX4* and downstream targets

**Figure 4:**
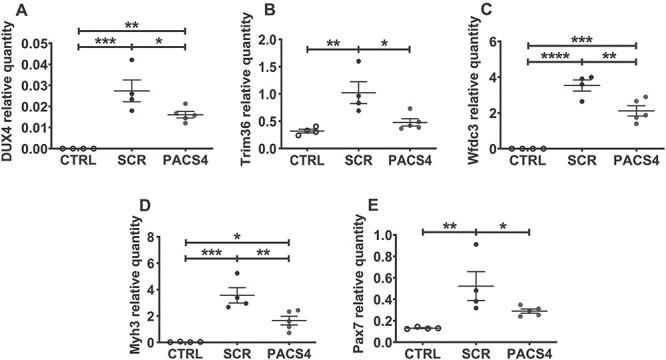
Vivo-PMO PACS4 treatment down-regulates expression of *DUX4* and relevant genes. Following TMX-induced *DUX4* expression and four weekly treatments with Vivo-PMOs, changes at mRNA levels in TA muscle were examined by RT-qPCR for *DUX4* (**A**), *Trim36* (**B**), *Wfdc3* (**C**), *Myh3* (**D**) and *Pax7* (**E**), relative to corresponding *Gapdh* expression. Data are shown as means ± SEM; *n* = 4–5. Statistical comparison was by one-way ANOVA followed by Tukey’s multiple comparisons test, *P* < 0.05 (^*^), *P* < 0.01 (^*^^*^), *P* < 0.001 (^*^^*^^*^), *P* < 0.0001 (^*^^*^^*^^*^).

DUX4-induced pathology of TA muscle has been extensively studied in numerous pre-clinical FSHD research (29,32,37–41). Characterization by the Jones Lab has also demonstrated that the level of *DUX4* transgene recombination in TA muscle was as high as in most muscles/organs examined (32). Hence, we focused our investigation on the effect of Vivo-PMO PACS4 treatment in this muscle type. To verify the antisense efficacy on mRNA expression, we carried out RT-qPCR quantification for *DUX4* and two downstream genes that have been previously demonstrated to be activated in FSHD animal models, *Trim43* and *Wfdc3* (Fig. 4A–C) (3,32,42). As predicted, the mRNA levels of all genes were greatly elevated in SCR group compared with the CTRL values. Treatment with PACS4 reduced mRNA quantities of the examined genes by about half of the SCR levels (*P* = 0.0453 for *DUX4*, *P* = 0.0146 for *Trim36* and *P* = 0.0063 for *Wfdc3*). Due to DUX4-induced muscle deterioration and subsequent muscle regeneration, we further assessed the mRNA levels of two genes indicative of muscle regeneration, *Myh3* (43) and *Pax7* (Fig. 4D and E) (44). Expression of both genes was up-regulated by 96-fold (*P* = 0.0052) and 4-fold (*P* = 0.0075), respectively, compared to CTRL levels. PACS4 significantly down-regulated the expression, relative to SCR values, by ~50% (*P* = 0.0093 for *Myh3* and *P* = 0.0461 for *Pax7*), confirming the antisense effect against *DUX4*- and FSHD-related genes.

### PACS4-mediated DUX4 inhibition greatly improves muscle histopathology

**Figure 5:**
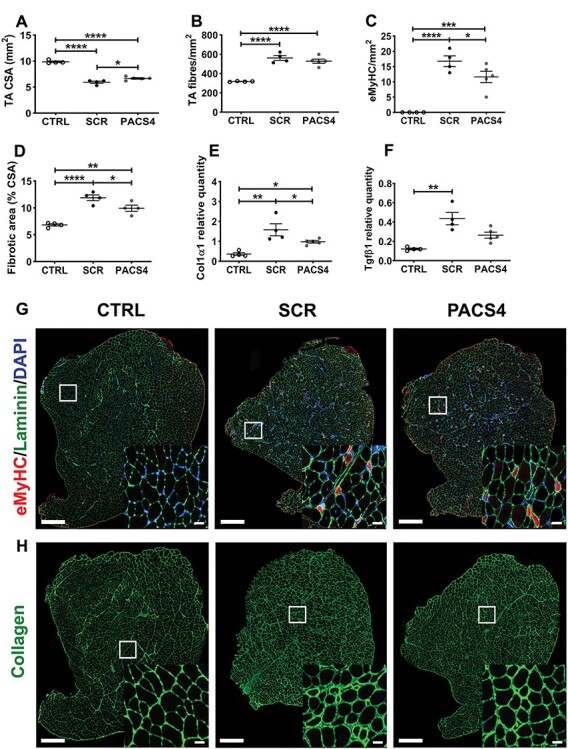
Effect of Vivo-PMO PACS4 therapy on muscle histopathology. Frozen TA muscle sections were stained for laminin, embryonic myosin heavy chain (eMyHC) and DAPI. The cross-sectional area (CSA) of the entire muscle section was automatically scored by MuscleJ (**A**). Laminin staining was used for identifying the fiber perimeter. An average of 3200 myofibers/TA was examined and displayed as the total number of myofibers (**B**) or the number of myofibers positive with eMyHC staining (**C**), per mm^2^ of the CSA. Fibrotic area in TA muscle was semi-automatically evaluated and expressed as percentage of the area positive for collagen of the muscle CSA (**D**). mRNA expression of gene indicative for fibrotic response, *Col1α1* (**E**) and *Tgfβ1* (**F**), was quantified by RT-qPCR. Statistical comparison was by one-way ANOVA followed by Tukey’s *post hoc* test (A–F). Data are shown as means ± SEM; *n* = 4–5; *P* < 0.05 (^*^), *P* < 0.01 (^*^^*^), *P* < 0.001 (^*^^*^^*^), *P* < 0.0001 (^*^^*^^*^^*^). Representative images of the entire TA cross-sections co-stained with eMyHC (red), laminin (green), DAPI (blue) (**G**), or single stained with collagen VI (**H**) are shown at magnification of ×100, scale bar = 500 μm. Corresponding enlarged images at higher magnification are shown in the subsets, scale bar = 50 μm (G, H).

To assess the effect of the treatment with Vivo-PMO PACS4 on muscle histopathology, TA muscle sections were immunostained for laminin to assist the identification of the myofiber sarcolemma for subsequent analyses of the cross-sectional area (CSA) and the number of total myofibers. We observed a decrease in the CSA of both MCM-D4 muscle groups, and that PACS4 treatment significantly protected the muscle from atrophy by 12%, relative to the SCR treatment (6.7 ± 0.2 mm^2^ versus 5.3 ± 0.2 mm^2^, *P* = 0.0268) (Fig. 5A). The number of TA myofibers per mm^2^ of the CSA in both MCM-D4 groups was significantly higher (*P* < 0.0001) than in the CTRL muscles (Fig. 5B). This consistently indicated muscle atrophy due to TMX-induced *DUX4* expression and further suggested myofiber turnover. The detection of high amounts of myofibers expressing embryonic MyHC (eMyHC), a marker of muscle regeneration (43), confirmed this hypothesis. Interestingly, the number of eMyHC-positive fibers was significantly less in TA receiving PACS4 (11.6 ± 1.9) than SCR (16.8 ± 1.8), *P* = 0.0401 (Fig. 5C), suggesting that PACS4 treatment reduced DUX4-mediated pathology. We additionally assessed gene expression of two cytokines associated with inflammation, *Il-10* and *Tnf-α*, and observed tremendous up-regulation in the levels of both genes relative to the CTRL values (Supplementary Material, Fig. S3). Although PACS4 treatment did not significantly suppress the level of either *Il-10* (*P* = 0.2072) or *Tnf-α* (*P* = 0.2133) as compared to SCR, reduced expression of these factors toward the CTRL levels was observed, indicating a potential benefit of PACS4 administration against muscle inflammation.

**Figure 6:**
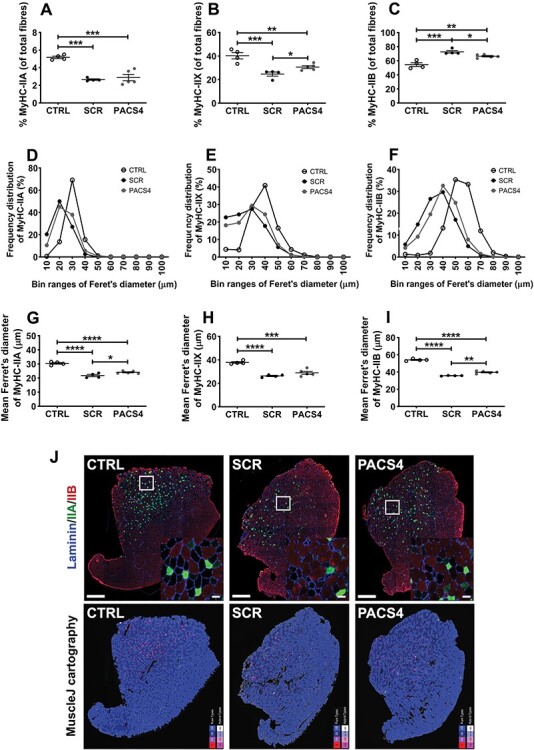
Vivo-PMO PACS4-mediated amelioration of muscle atrophy and myofiber type switching. Frozen TA muscle sections were immunostained for laminin (blue), MyHC IIA (green), MyHC IIB (red); unstained fibers were considered as MyHC IIX. The number of MyHC-positive fibers was automatically scored by MuscleJ and is expressed as the percentage of the total number of all myofibers within the entire muscle sections (**A**–**C**). Laminin staining was used for identifying the fiber sarcolemma and subsequent analysis of the minimal Feret’s diameter of myofibers. Histograms of frequency distribution (**D**–**F**) and the mean of the diameter of each myofiber type (**G**–**I**) are presented. Data are shown as mean ± SEM, *n* = 4–5. Statistical comparison was by one-way ANOVA and Tukey’s *post hoc* test (A–C, G–I); *P* < 0.05 (^*^), *P* < 0.01 (^*^^*^), *P* < 0.001 (^*^^*^^*^), *P* < 0.0001 (^*^^*^^*^^*^). Representative images of the entire TA muscle sections are shown at magnification of ×100, scale bar = 500 μm; enlarged images at higher magnification are shown in the subsets, scale bar = 50 μm. Corresponding cartographic images created by MuscleJ software are also displayed, with MyHC IIA, IIB and IIX fibers color-coded as purple, light blue and dark blue, respectively (**J**).

Further examination on the level of muscle fibrosis, based on immunostaining for a common marker of fibrosis, collagen VI (45,46), revealed that *DUX4* expression led to increase in the fibrotic area of TA muscle, from 6.82 ± 0.21% (CTRL) to 11.89 ± 0.54% (SCR, *P* < 0.0001). PACS4-treated muscle displayed 9.93 ± 0.57% fibrosis, a significant 17% reduction of the value in SCR muscle (*P* = 0.0403) (Fig. 5D). Since the level of fibrosis was up-regulated in MCM-D4 muscle, we additionally explored mRNA expression of genes indicative of a fibrotic response, *Col1α1* and *Tgfβ1* (Fig. 5E and F) (47)*.* As expected, *Col1α1* and *Tgfβ1* levels were higher in both SCR and PACS4 groups, compared to the level in CTRL. However, PACS4 treatment significantly reduced the mRNA expression, relative to that seen in SCR muscle, by 38% for *Col1α1* (*P* = 0.0421) and 40% for *Tgfβ1* (*P* = 0.0296), in agreement with the histological analysis for eMyHC and collagen VI (Fig. 5G and H). The consistency of our data describing muscle function, mRNA expression and histological improvement demonstrates that the TMX-induced DUX4 pathology used here is a reliable model of FSHD disease and that Vivo-PMO PACS4 administration inhibits DUX4 toxicity and decelerates the disease progression.

### Antisense treatment prevents a switch in myofiber types and improves myofiber atrophy

We initially assessed gene expression of four major myofiber types in mammalian skeletal muscles, including *Myh7* for slow-twitch MyHC I, *Myh2* for fatigue-resistant fast-twitch MyHC IIA, *Myh4* for fatigable fast-twitch MyHC IIB and *Myh1* for fast-twitch MyHC IIX (fatigue resistance less than IIA but better than IIB) (48). Expression of *Myh7* was undetectable using the analyses described in all TA groups suggesting that type I myofibers were not present, or sparse if they were, in the examined muscles. *Myh2* and *Myh1* levels in MCM-D4 muscle were lower than CTRL values by 60% (*P* = 0.0396) and 52% (*P* = 0.0160) in SCR-treated muscle and by 41% (*P* = 0.0742) and 43% (*P* = 0.0247) in PACS4-treated muscle, respectively (Supplementary Material, Fig. S4a and b). In contrast, *Myh4* expression was up-regulated by 141% (*P* = 0.0437) in SCR group, while treatment with PACS4 significantly reduced the level by 64% (*P* = 0.0213), toward the CTRL value (*P* = 0.4932) (Supplementary Material, Fig. S4c). We also performed immunostaining for laminin, MyHC IIA and IIB on TA transverse sections; unstained myofibers were considered as type IIX. Results from automatic MuscleJ quantification indicated a shift in myofiber populations, consistently with the qPCR analysis described above. MCM-D4 muscle displayed a significant decrease in the percentage of types IIA and IIX, but an increase in type IIB, as compared to CTRL groups (Fig. 6A–C). PACS4-treated muscle had similar quantity of type IIA relative to the SCR group (2.88% versus 2.65%, *P* = 0.8046). However, the percentage of MyHC IIX in TA receiving PACS4 was significantly higher than in SCR-receiving group (30.6% versus 24.7%, *P* = 0.0489) while the level of type IIB was respectively significantly lower by 8.5% (*P* = 0.0362), toward the CTRL property.

Further evaluation of the minimal Feret’s diameter of myofibers indicated myofiber atrophy occurring in all myofiber types of MCM-D4 mice (Fig. 6D–I), in line with the decrease in the CSA presented above. Histograms of the frequency distribution of the fiber size demonstrated that the majority of CTRL MyHC IIA, IIX and IIB fibers was 30, 40 and 50 μm in diameter, respectively, whereas the Feret’s diameter of corresponding MCM-D4 myofiber types peaked at 10-μm smaller values (Fig. 6D–F). Subsequent calculation of the mean fiber diameter clarified that the sizes of SCR-treated MyHC IIA, IIX and IIB fibers, relative to type-matched CTRL fibers, significantly decreased from 30.4 ± 0.6 to 21.8 ± 0.8 μm (*P* < 0.0001), 37.8 ± 0.7 to 26.3 ± 0.5 μm (*P* < 0.0001) and 54.1 ± 0.6 to 35.7 ± 0.2 μm (*P* < 0.0001), respectively. PACS4 did not alter the proportion of MyHC IIA, but significantly increased the myofiber diameter, compared to SCR treatment, from 21.8 ± 0.8 to 24.1 ± 0.4 μm (*P* = 0.0424). Administration of PACS4 was also effective in improving the mean fiber size of MyHC IIB, relative to SCR values, from 35.7 ± 0.2 to 39.7 ± 0.7 μm (*P* = 0.0014), Figure 6G–I. Representative images of immunostained TA sections analyzed by MuscleJ are shown in Figure 6J. Taken together, these histological analyses demonstrate that DUX4 expression induces muscle atrophy in TA muscle of MCM-D4 mice, in agreement with the reduction in the muscle mass and strength we detected. Treatment with Vivo-PMO PACS4 efficiently ameliorates DUX4 histopathology by preventing myofiber atrophy and shifts in the myofiber-type profile.

## Discussion

Despite increased understanding of genetic and epigenetic factors that contribute to FSHD, there is no treatment that can prevent or delay the disease progression. Clinical management involving physiotherapies, vision and hearing aids, orthopedic interventions, pain and fatigue management or surgical scapular fixation has shown some clinical benefit and improved the quality of life for FSHD patients (49,50). Since aberrant expression of the *DUX4* gene has been extensively reported as the main causative factor of FSHD (9–14,20), pre-clinical strategies silencing *DUX4* expression have shown promise for FSHD treatment (20,22,24,29,41,51–53). Among antisense approaches, those targeting the pLAM region of *DUX4* 3′UTR, including ours, have provided the best down-regulatory effect (20,24,29,30). As the stabilization of *DUX4* mRNA and translation of the protein require recognition of the polyadenylation signal (PAS) and the cleavage site (CS) in the pLAM region, we hypothesized and demonstrated in this study that antisense oligonucleotides (AONs), with phosphorodiamidate morpholino oligomer (PMO) chemistry, targeting both key elements enhanced the inhibitory efficacy of previous PMOs that target either the PAS or the CS (20,24,29). As demonstrated, newly designed PMOs, particularly the PACS4, were significantly more effective than the best PMO CS3 candidate described in the previous study (24), reducing mRNA expression of *DUX4* and its downstream genes to levels seen in isogenic positive control cells. These results clearly indicate that further optimization of the AON sequences can improve the impact of the antisense therapy. However, the antisense efficiency is dose dependent while *DUX4* expression is heterogeneous between FSHD-derived cell clones (33); therefore, more work is needed to verify that the optimized PMO PACS4 is effective in other patient *in vitro* models.

Although pre-clinical development of therapeutic approaches silencing *DUX4* gene has been promising, none has been translated into FSHD clinical testing. This is because most studies have been conducted in cell cultures. Several groups made further efforts, investigating the therapeutic benefit in FSHD animal models (29,30,41,52), but all studies so far have employed a local intramuscular administration. Optimizing a systemic therapy is clinically important because any treatment for FSHD will need to suppress *DUX4* expression in a large number of skeletal muscles. In addition, previous *in vivo* studies focused on assessment of the levels of DUX4 and downstream genes, with limited examination of the muscle function or histopathology improvement; hence, such investigations could not provide a complete pre-clinical evaluation of the therapeutic benefit of AON-mediated DUX4 suppression.

Following our *in vitro* optimization, we demonstrate here for the first time that systemic delivery of the optimal PMO PACS4 ameliorated DUX4-mediated pathology in an FSHD-like mouse model. We demonstrated increase in the mass of the examined skeletal muscles, improvement in the locomotor behavior and amelioration in the fatigue level of treated mice. The benefit provided to the tibialis anterior (TA) muscle was highlighted by the significant inhibitory effect on mRNA expression of *DUX4* and its targets, and the consequent beneficial effects on muscle regeneration and muscle fibrosis, and potentially on muscle inflammation. Most importantly, therapeutic outcome was demonstrated by enhancement of the muscle force generated and the decrease in the muscle atrophy. As no difference in the total myofiber number was detected, the increase in strength of treated muscles was likely due to the substantial changes in histological pathology as mentioned above and, at least partially, by antisense-mediated decrease in myofiber atrophy and prevention in the shift of myofiber populations from MyHC IIX to IIB. In FSHD, MyHC IIB fibers have been shown to generate significantly less force and display a decreased number of mitochondria than other fiber types (54,55). Therefore, we speculate that by suppressing the switch into MyHC IIB fibers our antisense treatment could provide additional beneficial effects in the mitochondrial content and function, and consequently in energy metabolism; clarification of this will need to be investigated further.

Despite these encouraging results, our study presents two issues that need to be investigated in future work. The first one is represented by the need of finding a safe system to allow efficient uptake of PMO chemistry. PMO has good stability in skeletal muscle (56,57) and is associated with no serious toxic concern in human, as seen in clinical trials for DMD (58,59). Nevertheless, as a charge-neutral chemistry, PMO displays limited cellular uptake, which means it does not easily penetrate muscles of FSHD patients. Conjugating PMO with a cell-penetrating moiety, for example the octaguanidine dendrimer used here, improves the cellular uptake and it is essential for FSHD therapies (60,61). As demonstrated, the antisense benefit of Vivo-PMO PACS4 can be achieved in TA muscle following systemic administration of the chemistry. Vivo-PMO further has enhanced stability due to its arginine-rich component (60), although this modification may cause unwanted effects (62). However, potential toxicity can be minimized through careful optimization of the dosage or studying the sensitivity of the organisms to be treated (28,61,63,64). For instance, the 10 mg/kg dose of Vivo-PMO used in this study has been proven to be effective with no detectable toxicity after six weekly systemic injections in the *PITX1* transgenic mouse model that is relevant to FSHD (61), but additional dose screening may maximize the antisense efficacy. PMO can be also conjugated with alternative cell-penetrating peptides (28,65–67) or with antibodies (68), or be delivered via nanoparticles (69) or even packaged into recombinant viral vectors (70), all of which have been shown to improve cell penetration and antisense efficacy in numerous pre-clinical studies. These alternative chemistries may be the future choice of antisense therapeutics, but their clinical safety and delivery mechanisms require further investigation.

The second issue is the animal model to be used. There are four animal models of FSHD commercially available, including the D4Z4–2.5 (42), iDUX4pA (47), FLExDUX4 (31) and Rosa26-DUX4 (38) mice. The investigators have greatly improved these models to recapitulate better the complicated pathophysiological mechanism of DUX4 activity and the phenotypic features seen in FSHD patients (32,71); nonetheless, a standard model of the disease has not been decided yet. This is an obvious obstacle for translational development of not only antisense therapies but also any approach for FSHD. The FLExDUX4 model we used here to generate double-transgenic MCM-D4 mice was chosen for its wide application in the FSHD research field (30,41,52); however, further refinement of this model may be needed. For example, *DUX4* expression following the specific TMX dose regimen we developed in this study was initially lower than the burst-like expression in the previous model (32). However, such TMX dosage overcame the innate muscle recovery and likely generated a more stable DUX4 pathology as similarly seen in other FSHD-like models (72). In addition, we chose to use only homozygous FLExDUX4 as they tend to display more consistent phenotype than heterozygous mice. We also studied only male mice to avoid potential intervention of estrogens in the readout because it has been shown to antagonize DUX4 toxicity in FSHD myoblast cultures (73), even though its clinical benefit remains under investigation (74). These applications may explain why the phenotype in our mice was less variable than previously reported (31,32) and the variance in the sample means was minor despite the small mouse number used. However, it is important to verify the antisense effect of Vivo-PMO PACS4 in female mice and use larger sample sizes to ensure the therapeutic benefit obtained here was not overestimated. Without TMX induction, leaky *DUX4* expression in the four single-transgenic models mentioned above can generate mild FSHD-like pathology and detectable muscle weakness with severity likely developed overtime. The non-induced models may therefore be suitable for studying the long-term effect of therapeutic applications for FSHD. In addition, because skeletal muscles are not equally affected in these models, consistently as seen in patients with FSHD (33,75), and DUX4 has been detected in biopsies of unaffected individuals (76), more research is needed to determine the level of DUX4 suppression required to achieve clinical therapeutic outcomes.

In summary, the present study provides substantial evidence that systemic *in vivo* treatment with an improved antisense design results in reduction in mRNA quantities of *DUX4* and target genes that leads to amelioration in the muscle function, muscle histopathology and locomotor activities of treated mice. Our data overall demonstrate that the optimal antisense approach can contribute to future development of a therapeutic strategy for FSHD.

## Materials and Methods

### PMOs and Vivo-PMOs

Phosphorodiamidate morpholino oligomers (PMOs) and Vivo-PMOs were purchased from GeneTools (Oregon, USA). To allow conjugation to octaguanidine dendrimer, 28-mer version of the optimized PMO was used in the *in vivo* work. PMOs and Vivo-PMOs were dissolved in sterile ddH_2_O and were further diluted to desired concentrations in cell culture medium (*in vitro* work) or in sterile 0.9% saline (Sigma, UK) immediately prior to injection into mice. Sequences of the PMOs and Vivo-PMOs are listed in Supplementary Material, Table S1.

### Cell cultures and PMO transfection

FSHD immortalized myoblast cells that have been characterized previously (33) were kindly provided by Dr Vincent Mouly, Institute of Myology, France. The A5 clone containing 3 D4Z4 units was considered as being contracted, while the A10 clone containing 13 D4Z4 units was considered as being non-contracted and used as a positive control. Cells were maintained in proliferation medium composed of 64% (v/v) high glucose DMEM (Gibco, UK), 16% (v/v) Medium 199 (Gibco, UK), 20% (v/v) fetal bovine serum (FBS, Gibco, UK), 50 μg/ml gentamicin (Sigma, UK), 0.2 μg/ml dexamethasone (Sigma, UK), 0.5 ng/ml human basic fibroblast growth factor (Sigma, UK), 5 ng/ml human recombinant epidermal growth factor (Sigma, UK) and 25 μg/ml fetuin from FBS (Sigma, UK). Cell differentiation was induced when cells reached around 90% confluence by replacing the proliferation medium with 99% (v/v) high glucose DMEM (Gibco, UK), 1% (v/v) horse serum (Gibco, UK) and 10 μg/ml human insulin–transferrin–sodium selenite media supplement (Sigma, UK). To study the antisense inhibitory effect, myoblasts were differentiated for 2 days and treated with 1 or 10 μm PMOs, *n* = 3 per cell group. Transfection was facilitated by 6 μm Endo-Porter (GeneTools, Oregon, USA). RNA extraction was conducted after two additional days.

### RT-qPCR quantification for *DUX4* and relevant genes

Total RNA from cultured cells was extracted using RNeasy kit (QIAgen, UK), while RNA from murine muscles (as described in the animal study below) was extracted using RNeasy Fibrous Tissue kit (QIAgen, UK), following the manufacturer’s instructions. Tissue homogenization was performed in the lysis buffer provided with the kit, at 25 Hz for 2–4 min, on a TissueLyser II (QIAgen, UK). RNA was quantified on an ND-1000 NanoDrop spectrophotometer (Thermo Scientific, UK). One microgram RNA was reverse transcribed using QuantiTect reverse transcription kit (QIAgen, UK). Ten nanograms of diluted cDNA in qPCR water (Roche, UK) were then amplified using LightCycler480 SYBR Green Master I kit (Roche, UK), according to the manufacturer’s instructions; samples were prepared in triplicates. Reactions were run on LightCycler480 System, initialized at 95°C for 5 min, followed by 45 cycles at 95°C for 15 s, 58^o^–62°C for 15 s and 72°C for 15 s. Relative quantification for *DUX4* or its relevant genes was performed against corresponding housekeeping genes, *B2M* or *Gapdh*. Primers were purchased from Integrated DNA Technologies (Belgium) and are detailed in Supplementary Material, Table S3.

### Immunocytochemistry for quantifying cell fusion index

FSHD immortalized myoblast cells were seeded in 6-well plates pre-coated with extracellular matrix gel from Engelbreth-Holm-Swarm murine sarcoma (Sigma, UK). Following transfection with PMOs, the culture medium (as described above) was removed on day 4 of cell differentiation. Cells were rinsed in ice cold 1× PBS (Sigma, UK), fixed in ice cold 4% (w/v) paraformaldehyde (Sigma, UK) for 10 min and permeabilized in 1× PBST (0.25% (v/v) Triton X-100, 1× PBS, Sigma, UK) for 10 min. Cells were blocked in 1% (w/v) bovine serum albumin, BSA (Sigma, UK), 10% (v/v) goat serum (Sigma, UK), 1× PBST for 1 h. Incubation with mouse antimyosin heavy chain (MF20) primary antibody (1:100, DSHB, Iowa, USA) was performed at 4°C overnight, followed by incubation with goat antimouse AlexaFluor488 secondary antibody (1:500, Life Biotechnologies, UK) for 1 h at room temperature. MF20 antibody was generated and deposited to DSHB by Fischman (77). Nuclei were further stained with 1 μg/ml 4′,6-diamidino-2-phenylindole, DAPI (Sigma, UK) in 1× PBS for 10 min. Cells were kept in 1× PBS at 4°C until the cell images were visualized on an inverted fluorescence Axio Observer D1 microscope (Zeiss, UK). Four random image fields from each cell culture well were captured at a magnification of ×100 by an AxioCam MR3 combined with ZEN Imaging software (Zeiss, UK). The cell fusion index was then evaluated as the number of nuclei in MF20-positive myotubes containing ≥3 nuclei and expressed as the percentage of the total nuclei number in the image field.

### Animals

Animals were bred in a minimal disease facility at Royal Holloway University of London, with food and water *ad libitum*. FLExDUX4 (JAX 028710) and HSA-MCM (JAX 025750) mice were purchased from The Jackson Laboratory (Maine, USA). FLExDUX4 colony was maintained as homozygous for Gt (ROSA)26Sor^tm1.1(DUX4^*^)Plj^, while HSA-MCM colony was maintained as hemizygous for Tg (ACTA1-cre/Esr1^*^)2Kesr. Tamoxifen (TMX)-inducible Cre-driver FLExDUX4 bitransgenic line (aka. MCM-D4) used in this study was generated by crossing FLExDUX4 females with HSA-MCM males. Due to gender specific–DUX4 pathology in the MCM-D4 model, only males were used and littermates were allocated equally between groups. All mice were kept under a standard 12-h light/dark cycle and were supervised on a daily basis by experienced animal staff.

### *In vivo* experimental design

In the initial study optimizing tamoxifen (TMX) dosage for inducing constant *DUX4* expression, 16-week-old MCM-D4 mice received either a single dose of 5 mg/kg TMX (*n* = 5) or 2.5 mg/kg TMX once every 2 weeks (*n* = 5) via intraperitoneal (IP) injection. TMX (Sigma, UK) was prepared as described previously (31) and diluted in warmed sterile corn oil to 1 mg/ml prior to use. HSA-MCM mice, considered as wild-type control (*n* = 5), received volume-match corn oil. Bodyweight was recorded weekly. *In situ* TA force measurement was performed at week 4, while mice were under terminal anesthesia prior to TA muscle dissection.

In the subsequent study evaluating antisense therapeutic effect, 16-week-old MCM-D4 males were IP injected with 2.5 mg/kg TMX on days 0 and 14 to induce *DUX4* expression. HSA-MCM mice receiving the same TXM dosage were considered as wild-type control (*n* = 4). MCM-D4 mice were further IP injected with 10 mg/kg of Vivo-PMO PACS4 (*n* = 5), or 10 mg/kg of Vivo-PMO SCR (*n* = 4), considered as a negative control, on days 2, 8, 16 and 22, while HSA-MCM mice received volume-matched saline. Animals underwent treadmill exhaustion tests (days −6, 11 and 25) and locomotor behavioral tests (days 22, 25 and 28) prior to being put under terminal anesthesia for *in situ* TA force measurement and subsequent tissue collection (day 30). Mice were kept under isoflurane-induced anesthesia (3% in 100% CO_2_) during injections and were continuously monitored until they fully recovered and then hourly for 3 h post-recovery.

### Treadmill exhaustion test

All tests were performed on a Treadmill Simplex II system (Columbus Instruments, Ohio, USA), with adjusted 15°C inclination. Mice were acclimatized to the apparatus for 5 min on an unmoving treadmill and at a speed of 5 m/min for additional 5 min. The speed was then increased by 0.5 m every minute. Mice were exercised until they were unable to remain off the stopper for 10 s. Total running time was recorded and displayed as time to fatigue as percentage of the baseline time recorded on day −6.

### Open-field locomotor activity

Mouse open-field behavioral activity was examined using locomotor activity monitors as previously described (56). Mice were acclimatized to the test chamber during an undisturbed period of 15 min before the data were acquired and collected by Amon Lite software (version 1.4) every 10 min in a 60-min session. Data obtained from each mouse were averaged. The same procedure was repeated every 3 days for three times. During the acquisition, particular care was taken to minimize noise and movement into the room. Both the locomotor activity monitors and the software were obtained from Linton Instrumentation, UK.

### *In situ* muscle force measurement

Mice were anesthetized by IP injection with a mixture of 10 mg/ml dolethal (Vetoquinol, UK) and 15 μg/ml buprenodale (Dechra, UK) at five times of the bodyweight. The distal tendon of tibialis anterior (TA) muscle was dissected and attached to an isometric transducer, Dual-mode muscle lever (Aurora Scientific, Canada), through a loop made of braided silk suture (Harvard Apparatus, UK). The sciatic nerve was isolated and distally stimulated by a bipolar silver electrode using supramaximal square wave pulses at 0.1 ms duration. Data provided by the isometric transducer were recorded and analyzed using Dynamic Muscle Control and Analysis Software (Aurora Scientific, Canada). All isometric measurements were obtained at an initial length at which a maximal tension was recorded during the tetanus. Responses to tetanic stimulations at increased pulse frequencies from 10 to 180 Hz were recorded and the maximal force (mN) was determined. The specific force (mN/mm^2^) was subsequently calculated based on a ratio of the maximal force and the muscle cross-sectional area (CSA) that was approximated mathematically by dividing the muscle mass by the optimum fiber length and the density of mammalian muscle, as described in TREAT-NMD SOP DMD_M.2.2.005.

### Post-mortem tissue processing

From each mouse, the gastrocnemius (GAS), quadriceps (QUAD) and TA muscles were collected. Tissues from one side of hindlimb were frozen immediately in liquid nitrogen for RNA extraction (as described above). Contralateral muscles were embedded in optimal cutting temperature medium (VWR, UK) and subsequently frozen in liquid nitrogen–cooled isopentane (Sigma, UK) and stored at −80°C. Frozen TA muscle was cryosectioned on an OTF5000 cryostat (Bright, UK) at 10-μm thickness for 10 serial levels through the muscle length, and transverse sections were collected onto SuperFrost slides.

### Immunohistochemistry staining

For collagen immunostaining, serial muscle section–containing slides were fixed in ice-cold acetone for 10 min and blocked in 1% (w/v) BSA, 1% (v/v) goat serum, 0.1% (v/v) Triton X-100 and 1× PBS for 1 h. Subsequent incubation with rabbit anticollagen VI (1:300, Abcam, UK) antibodies was carried out at 4°C, overnight. Slides were washed three times in 1× PBS, 0.05% (v/v) Tween-20 prior to 1-h incubation with goat antirabbit AlexaFluor488 (1:1000, Life Biotechnologies, UK). An additional incubation for 15 min with 1 μg/ml DAPI was performed before slides were mounted in Mowiol 4–88. Reagents were purchased from Sigma, UK, unless stated otherwise. Images from the largest mid-belly muscle sections were captured on Axio Observer D1 fluorescence microscope (Zeiss, UK) at a magnification of ×100 by an AxioCam MR3 and were automatically stitched together by ZEN Imaging software (Zeiss, UK) to generate an image of the whole transverse muscle section.

For laminin and myosin heavy chain (MyHC) co-immunostaining, frozen sections were fixed in ice-cold acetone for 10 min and then blocked in mouse-on-mouse blocking buffer (Vector Laboratories, UK) supplemented with 1% (w/v) BSA, 1% (v/v) goat serum, 0.1% (v/v) Triton X-100 and 1× PBS for 30 min. Subsequent incubation with primary antibodies was carried out at 4°C for overnight, and then with compatible secondary antibodies for 1 h, at room temperature. Primary antibodies were rabbit antilaminin antibody (1:300, Abcam, UK) and mouse anti-MyHC antibodies (DSHB, Iowa, USA), including SC-71 and BF-F3 for fast-twitch fiber types IIA and IIB, respectively (1:5), BF-G6 for embryonic MyHC (1:50), unstained fibers were considered as IIX. All DSHB antibodies were deposited by Schiaffino Secondary antibodies (Life Technologies, UK) were goat antirabbit IgG Alexa405 (1:400) or goat antirabbit IgG Alexa488 (1:400), goat antimouse IgG2b Alexa488 (1:400), goat antimouse IgM Alexa568 (1:250) and goat antimouse IgG Alexa568 (1:400), respectively. Nuclei were stained with 1 μg/ml DAPI. Slides were mounted in Mowiol 4–88 (Sigma, UK). Images of whole transverse muscle sections were acquired and generated as described above.

For hematoxylin and eosin staining, frozen muscle sections were fixed in 100% (v/v) ice-cold methanol for 10 min and then submerged in hematoxylin (Vector Laboratories, UK) and eosin. Sections were dehydrated in a series of ethanol washes of 50%, 80%, 100% (v/v), 1 min/wash, cleared in 100% (v/v) xylene for 2 × 5 min. Slides were mounted in DPX mountant. Reagents were purchased from Sigma, UK, unless stated otherwise. Images from each of mid-belly muscle sections were captured using an Eclipse Ni-E upright microscope and compatible software (Nikon Instruments Inc., New York, USA).

### Histological analyses

Laminin staining was used for identifying the fiber perimeter. The number of total myofibers, MyHC-IIA, -IIB or -IIX fibers, as well as the minimal Feret’s diameter of individual fibers, was automatically measured by MuscleJ software (National Institutes of Health, Maryland, USA), while the number of eMyHC was counted manually. Automatic analysis of the frequency distribution of the minimal Feret’s diameter was carried out using GraphPad Prism6 software (California, USA). The CSA of the entire muscle sections or the area positive with collagen staining was semi-automatically scored by MuscleJ software. Fibrotic area was calculated as the percentage of the total area of the muscle cross-section.

### Statistical analysis

Data were analyzed using GraphPad Prism8 software (California, USA) and are shown as the means ± SEM. Error bars represent the S.E.M; ‘*n*’ refers to the number of biological replicates in cell culture work or the number of mice per group. All data passed the normality Shapiro–Wilk test, which is the most powerful test among four common normality tests especially for small sample size (3 ≤ *n* ≤ 5000) (78). Comparisons of statistical significance were further assessed by one-way or two-way ANOVA followed by Tukey’s *post hoc* test, as detailed in figure legends. All functional tests and histological analysis were performed in a blinded manner.

## Supplementary Material

Lu-Nguyen_et_al_Antisense_therapy_for_FSHD_Revised_supplemental_data_ddab136
