# Supplementary material for: Systemic antisense therapeutics inhibiting DUX4 expression ameliorates FSHD-like pathology in an FSHD mouse model
Source: Hum Mol Genet. 2021 May 13;30(15):1398–412. doi: 10.1093/hmg/ddab136 (PMC8283208; doi:10.1093/hmg/ddab136)
Supplement: Lu-Nguyen_et_al_Antisense_therapy_for_FSHD_Revised_supplemental_data_ddab136 [file lu-nguyen_et_al_antisense_therapy_for_fshd_revised_supplemental_data_ddab136.pdf]

Supplemental data

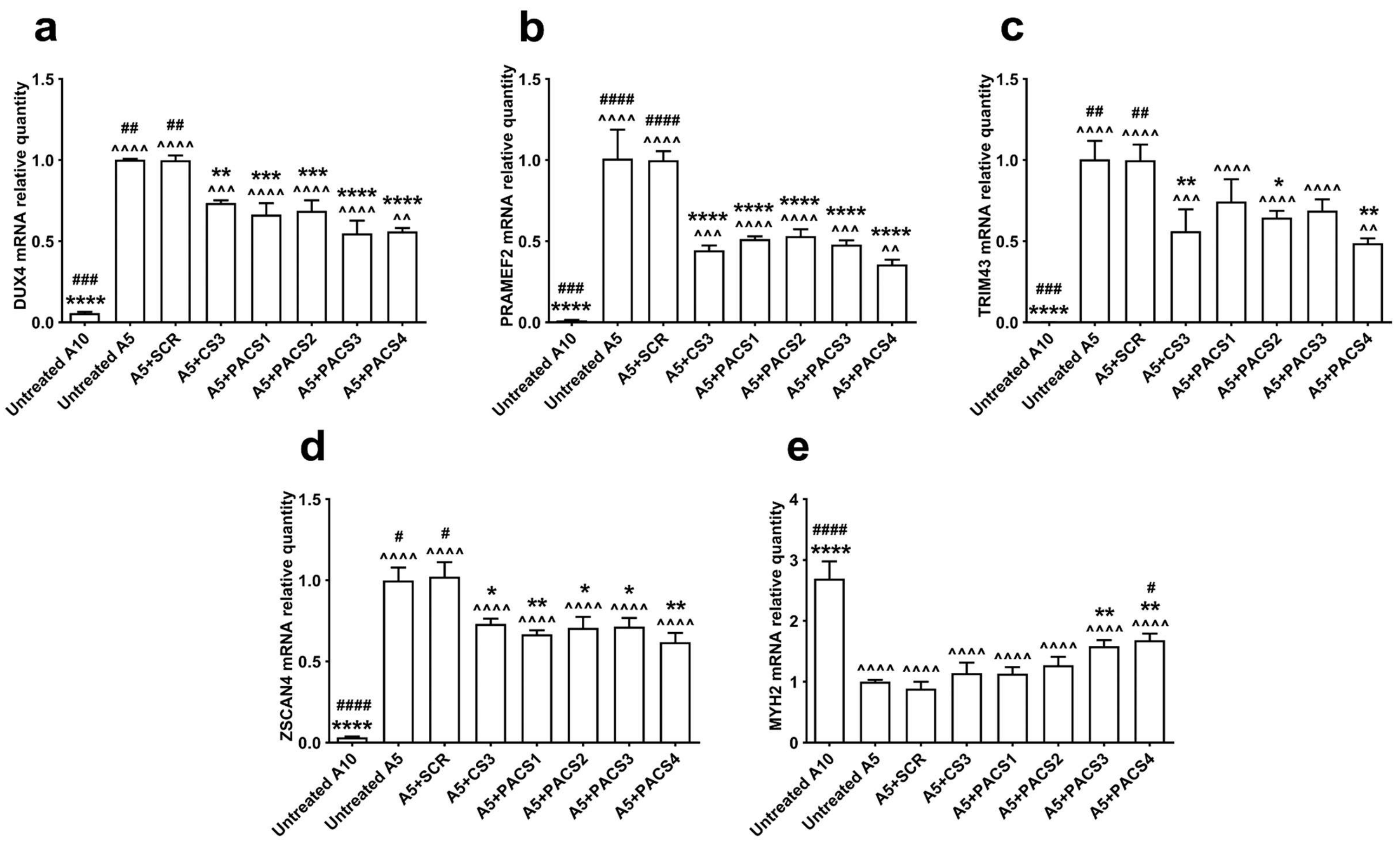

Figure S1: Effect of 1 µM PMO treatment in immortalized FSHD myoblast cell cultures. Immortalized A5 myoblasts were differentiated for 2 days before the cells were treated with 1 µM PMOs via Endo-Porter-mediated transfection. Immortalized A10 or A5 cells receiving only Endo-Porter reagent were considered as untreated positive or negative control, respectively. Total RNA was extracted 2 days after PMO treatment. RT-qPCR quantification for *DUX4* **(a)**, *PRAMEF2* **(b)**, *TRIM43* **(c)**, *ZSCAN4* **(d)**, and *MYH2* **(e)** are shown. Statistical comparison was by one-way ANOVA followed by Tukey's multiple comparisons test. Carets, asterisks or hashes indicate significances compared with untreated A10, untreated A5 or A5 treated with PMO CS3, respectively. Data are shown as mean ± S.E.M., $n = 3$, *$p < 0.05$, **$p < 0.01$, ***$p < 0.001$, ****$p < 0.0001$.

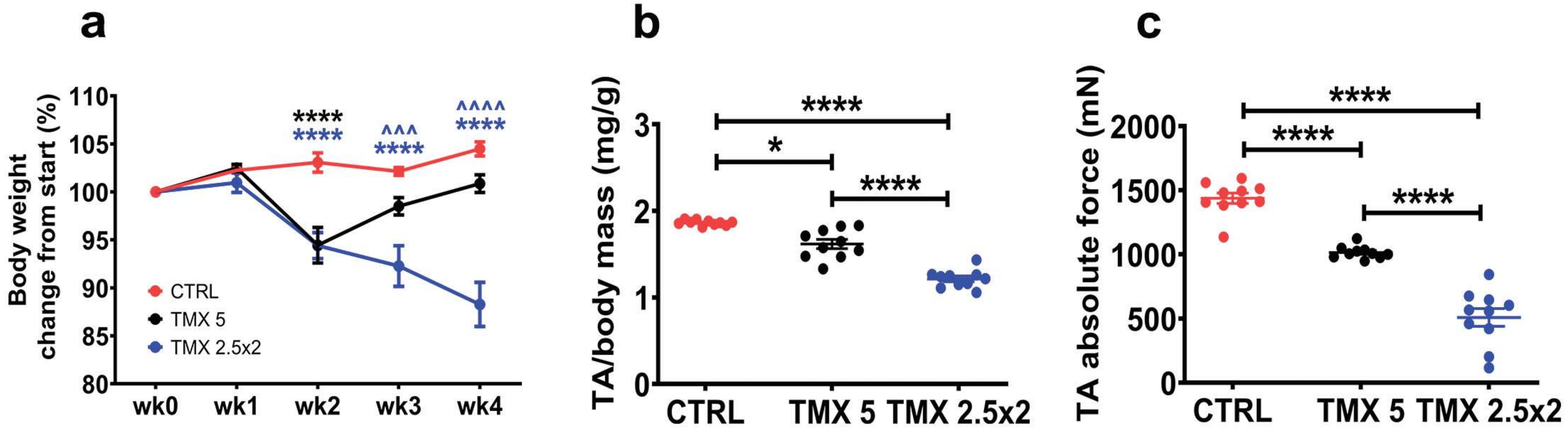

Figure S2: Optimizing tamoxifen (TMX) dosage for inducing progressive DUX4 pathology in double transgenic MCM-D4 mice. Male MCM-D4 mice received either a single dose of 5 mg/kg TMX (TMX 5) at week 0 or 2.5 mg/kg/biweekly TMX (TMX 2.5x2) at weeks 0, 2 via intraperitoneal injection. A group of HSA-MCM mice receiving volume-matched corn oil was considered as control (CTRL). Body weight recorded weekly is displayed as the percentage of weight change from initial **(a)**. The mass of tibialis anterior (TA) muscle on both sides of the body normalized to the body weight is shown **(b)**. At week 4, mice were put under terminal anesthesia and the absolute force of both TAs from each mouse were assessed **(c)**. Statistical comparison was by one-way (b) or two-way (a, c) ANOVA followed by Tukey's multiple comparisons test. Data are shown as mean ± S.E.M., $n = 5$, *$p < 0.05$, **$p < 0.01$, ***$p < 0.001$, ****$p < 0.0001$. In **(a)**, black asterisks indicate significance between TMX 5 and CTRL groups; blue asterisks or carets indicate significant between TMX 2.5x2 and CTRL or TMX 2.5x2 and TMX 5 groups, respectively.

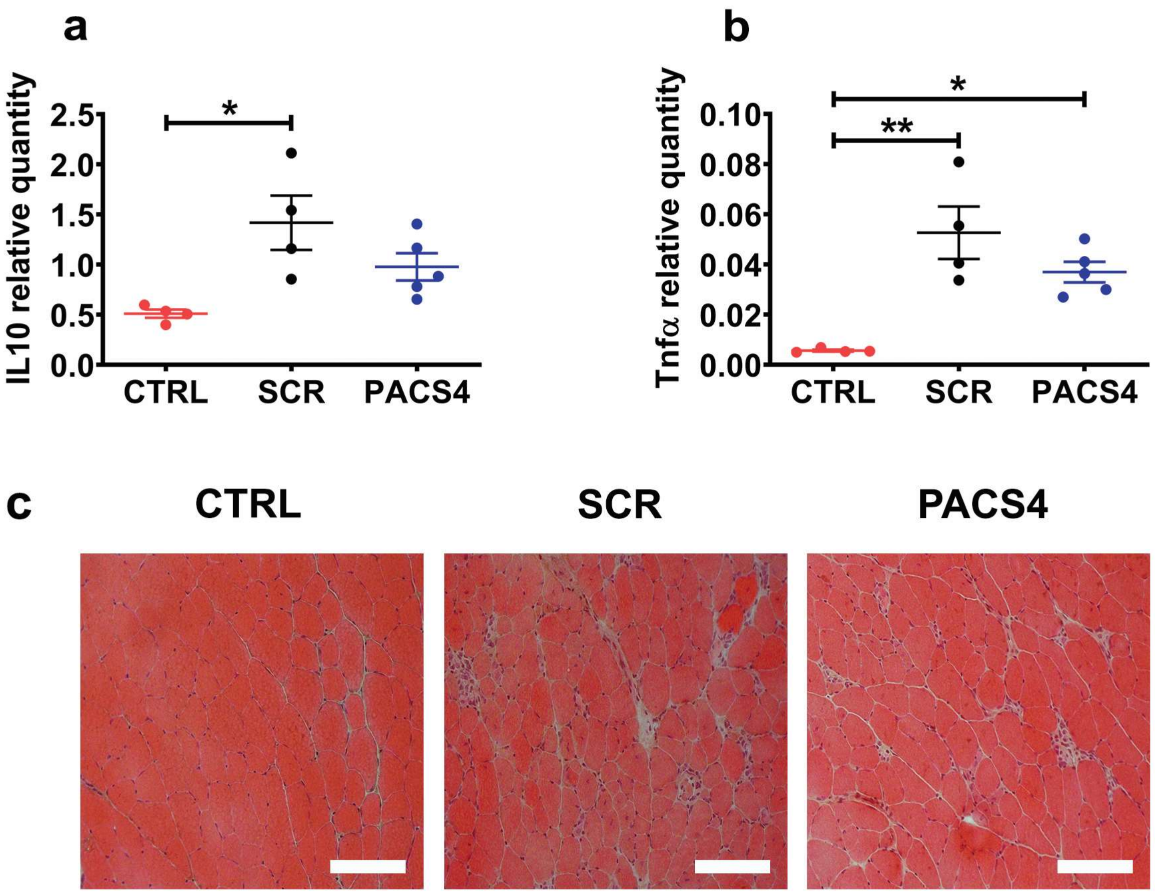

Figure S3: Effect of PACS4 treatment on muscle inflammation. mRNA levels of two inflammatory indicators were assessed by RT-qPCR **(a, b)**. Data are shown as means ± S.E.M; $n = 4$-5. Statistical comparison was by one-way ANOVA followed by Tukey's *post-hoc* test; $*p < 0.05$, $**p < 0.01$. Frozen TA muscle sections were stained with hematoxylin and eosin; representative images are shown at magnification of x100, scale bars = 100 µm **(c)**.

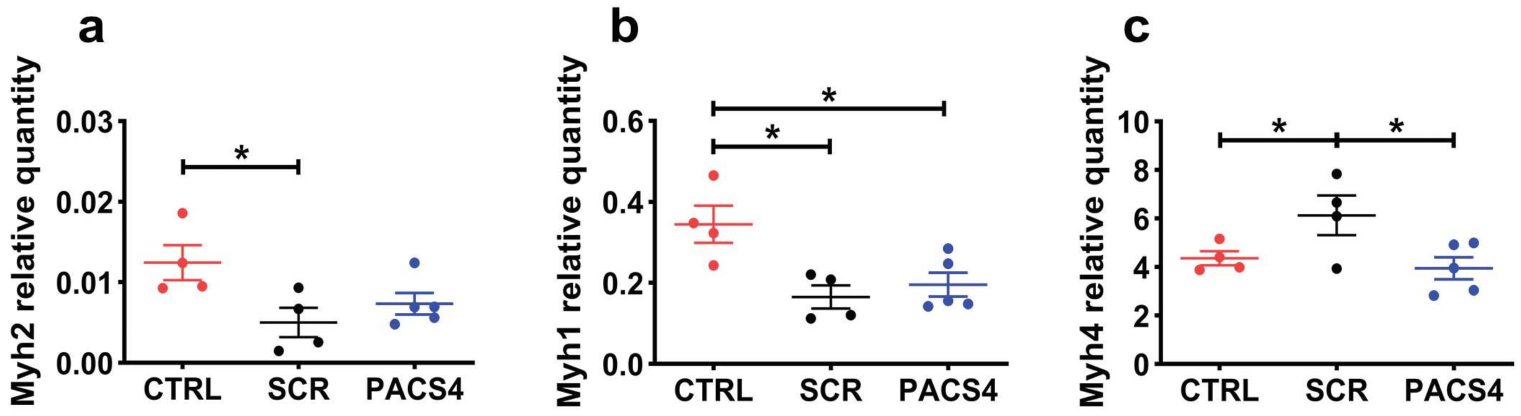

Figure S4: Gene expression of major myofiber types in TA muscle. mRNA levels of four fiber types were assessed by RT-qPCR, including *Myh2* for MyHC IIA **(a)**, *Myh1* for MyHC IIX **(b)**, and *Myh4* for MyHC IIB **(c)**, relative to corresponding *Gapdh* expression. *Myh7* for MyHC I was undetectable. Data are shown as means ± S.E.M; $n = 4\text{-}5$. Statistical comparison was by one-way ANOVA followed by Tukey's *post-hoc* test; $^*p < 0.05$.

Table S1: Sequences of PMOs and targeting regions within *DUX4* 3'UTR

**Name**	**PMO sequence (5'-3')**	**Target sequence (5'-3')**
PMO SCR, 25-mer	CCTCTTACCTCAGTTACAATTTATA	
PMO CS3, 30-mer (+2 +31)	TATAGGATCCACAGGGAGG**A**GGCATTTTAA	TTAAAATGCC**C**CCTCCCTGTGGATCCTATA
PMO PACS1, 30-mer (-2 +28)	AGGATCCACAGGGAGGGGGCATTTTAATAT	ATATTAAAATGCCCCCTCCCTGTGGATCCT
PMO PACS2, 30-mer (-1 +29)	TAGGATCCACAGGGAGGGGGCATTTTAATA	TATTAAAATGCCCCCTCCCTGTGGATCCTA
PMO PACS3, 30-mer (-2 +28)	AGGATCCACAGGGAGG**A**GGCATTTTAATAT	ATATTAAAATGCC**C**CCTCCCTGTGGATCCT
PMO PACS4, 30-mer (-1 +29)	TAGGATCCACAGGGAGG**A**GGCATTTTAATA	TATTAAAATGCC**C**CCTCCCTGTGGATCCTA
PMO PACS4, 28-mer (+1 +28)	AGGATCCACAGGGAGG**A**GGCATTTTAAT	ATTAAAATGCC**C**CCTCCCTGTGGATCCT

Polyadenylation signal (PAS): TTTAAT (PMO sequence) → ATTAAA (target sequence)

Cleavage site (CS): AGGATC (PMO sequence) → GATCCT (target sequence)

Modified nucleotide: **A** (PMO sequence) → **C** (target sequence)

Table S2: Changes in mouse locomotor behavior after 4 weeks of Vivo-PMO treatment

Open-field cage activity		WT		SCR		PACS4		SCR vs WT	PACS4 vs WT	PACS4 vs SCR
**Parameters**	**Descriptions**	**Mean**	**SEM**	**Mean**	**SEM**	**Mean**	**SEM**	***p* value**	***p* value**	***p* value**
Total activity	Total beam breaks	1633.2	132.6	267.5	55.0	702.2	79.2	< 0.0001	< 0.0001	< 0.0001
Fast activity	Fast beam breaks	185.7	24.3	26.6	8.1	35.4	5.3	0.1638	0.2596	0.9747
Slow activity	Slow beam breaks	1447.5	118.8	240.9	47.6	666.8	74.7	< 0.0001	< 0.0001	< 0.0001
Total static counts	Total beam breaks (movement lower than mobile threshold)	1233.0	93.4	222.6	40.0	629.2	71.6	< 0.0001	< 0.0001	< 0.0001
Fast static counts	Beam breaks (movement lower than mobile threshold and faster than fast threshold)	62.5	9.4	8.1	1.6	18.9	3.3	0.9601	0.9656	0.9909
Slow static count	Beam breaks (movement lower than mobile threshold and slower than fast threshold)	1170.5	87.9	214.5	39.1	610.3	68.9	< 0.0001	< 0.0001	< 0.0001
Total mobile counts	Total beam breaks (movement greater than mobile threshold)	400.2	47.1	44.9	19.6	73.0	12.1	< 0.0001	< 0.0001	0.9116
Fast mobile counts	Beam breaks (movement greater than mobile threshold and faster than fast threshold)	123.2	16.3	18.5	7.8	16.5	2.5	0.4654	0.4405	0.9985
Slow mobile counts	Beam breaks (movement greater than mobile threshold and slower than fast threshold)	277.0	36.2	26.4	11.9	56.5	9.7	0.0011	0.0026	0.9502
Total rearing counts	Number of rearing beam breaks	508.5	45.1	38.9	10.1	96.3	13.5	< 0.0001	< 0.0001	0.032
Fast rearing counts	Number of fast rearing beam breaks	241.6	26.1	25.3	7.3	47.0	7.3	0.0075	0.0107	0.9307
Slow rearing counts	Number of slow rearing beam breaks	266.9	27.0	14.4	3.1	49.3	6.9	0.0036	0.0279	0.9998
Total centre rearing counts	Number of rearing beam breaks occurred away from the cage walls	85.0	13.0	3.9	1.4	10.1	1.8	0.7508	0.8459	0.9985
Fast centre rearing counts	Number of fast rearing beam breaks occurred away from the cage walls	36.1	4.9	2.6	1.1	4.1	1.1	0.8722	0.9566	> 0.9999

Slow centre rearing counts	Number of slow rearing beam breaks occurred away from the cage walls	48.2	9.7	1.0	0.4	6.0	0.9	0.8722	0.9566	0.9995
Active time	Time of mobile or static activity (sec)	1153.5	86.4	215.4	41.3	584.5	64.0	< 0.0001	< 0.0001	< 0.0001
Static time	Time of static activity (sec)	943.8	68.1	189.7	33.7	538.5	59.7	< 0.0001	< 0.0001	< 0.0001
Mobile time	Time of mobile activity (sec)	209.7	24.1	25.8	10.3	45.9	7.1	< 0.0001	< 0.0001	0.9589
Rearing time	Time spent rearing (sec)	947.4	92.9	59.9	14.3	182.6	18.1	< 0.0001	< 0.0001	0.0161
Front to back counts	Number of traverses from front to back	115.2	11.4	18.3	5.2	35.5	5.7	< 0.0001	< 0.0001	0.0408
Inactive time	Time spent in inactivity (sec)	2446.5	86.4	3384.6	41.3	3015.5	64.0	< 0.0001	< 0.0001	< 0.0001
Distance travelled meters	Total distance travelled (m)	72.8	6.0	14.6	3.1	31.8	3.6	< 0.0001	< 0.0001	0.0238

Data were assessed by GraphPad Prism8 (California, USA). Statistical significance was analyzed by one-way ANOVA followed by Tukey's *post-hoc* test.

Table S3: Details of qPCR primers

Target gene	Accession number	Primer sequence (5'-3')	Amplicon size (bp)	Annealing temp (ºC)
B2M	NM_004048	Forward: CTCTCTTTCTGGCCTGGAGG<br>Reverse: TGCTGGATGACGTGAGTAAACC	67	60
DUX4 3'UTR	Gene ID: 100288687	Forward: CTCTGTGCCCTTGTTCTTC<br>Reverse: TCCAGGAGATGTAACTCTAATCCA	98	60
MYH2	NM_017534	Forward: TCAGGTCTTCCCCATGAACC<br>Reverse: GCTTATACACAGGCAGCCAC	185	60
PRAMEF2	NM_023014	Forward: ACCTTCTTCAGTGGGCACCT<br>Reverse: TGGGAACTGGGAGAGACACT	120	60
TRIM43	NM_138800	Forward: ACCCATCACTGGACTGGTGT<br>Reverse: CACATCCTCAAAGAGCCTGA	100	60
ZSCAN4	NM_152677	Forward: CTGGAGCAGTTTATGATTGG<br>Reverse: AGCTTCCTGTCCCTGCATGT	162	60
Col1α1	NM_007742	Forward: GAAACTTTGCTTCCCAGATGTC<br>Reverse: AGACCACGAGGACCAGAA	94	58
Gapdh	NM_008084	Forward: TCCATGACAACTTTGGCATTG<br>Reverse: TCACGCCACAGCTTTCCA	103	60
Il-10	NM_010548	Forward: CTTACTGACTGGCATGAGGATCA<br>Reverse: GCAGCTCTAGGAGCATGTGG	101	60
Myh1	NM_030679	Forward: GTGGAAGCTATCAAGGGTCTGC<br>Reverse: TCTTGCGGTCTTCCTCAGTTTG	79	60
Myh2	NM_001039545	Forward: ACCCTCTTATTTCCCAGCTGCAC<br>Reverse: ACTGCTGAACTCACAGACCCTTAC	61	60
Myh3	NM_001099635	Forward: ACCTCTAGCCGGATGGT<br>Reverse: AATTGTCAGGAGCCACGAAAAT	103	60
Myh4	NM_010855	Forward: AGAAACTGGAGGCTAGGGTGAG<br>Reverse: TCGTGCTTACGAAGACCCTTGAC	94	60
Myh7	NM_001361607	Forward: ATACGCATGCTTGTGCCGTAGG<br>Reverse: TTCCTTTCTCGGAGCCACCTTG	65	60
Pax7	NM_011039	Forward: CTCAGTGAGTTCGATTAGCCG<br>Reverse: AGACGGTTCCCTTTGTCGC	144	60

Tgfβ1	NM_011577	Forward: TGACGTCACTGGAGTTGTACGG<br>Reverse: TCGAAAGCCCTGTATTCCGTCTC	61	62
Tnf-α	NM_013693	Forward: AGCCGATGGGTTGTACCTTG<br>Reverse: ATAGCAAATCGGCTGACGGT	99	58
Trim36	NM_178872	Forward: TGAAAGTGGGAGTTGCTTCC<br>Reverse: GAATCAAAACAGGCGTCCTC	127	60
Wfdc3	NM_027961	Forward: CTTCCATGTCAGGAGCTGTG<br>Reverse: ACCAGGATTCTGGGACATTG	134	58
